# Spatial disparities in access to NHS dentistry: a neighbourhood-level analysis in England

**DOI:** 10.1093/eurpub/ckae099

**Published:** 2024-06-22

**Authors:** Stephen D Clark

**Affiliations:** School of Geography and Consumer Data Research Centre, University of Leeds, Leeds, United Kingdom

## Abstract

Over the past decade, access to National Health Service (NHS) dentistry in England has been problematic. There are increasing media reports of patients being unable to find treatment at a local NHS dentist. However, the extent of this issue varies by location and by the characteristics of the neighbourhood. The study uses official data sources on NHS dental provision and population. Travel accessibility is measured using car journey times. An advanced form of Floating Catchment Area accessibility is used, which accounts for supply competition, varying catchments, and distance decay. Spatial availability and accessibility indices are calculated. Ways in which the method can be used to explore various types of ‘what-if’ scenarios are outlined. Both availability and accessibility vary by the level of neighbourhood deprivation and the urban/rural nature of the neighbourhood. A case study, based on a real-world situation, shows the impact on the local neighbourhood of the closure of a dental practice. For all neighbourhoods, NHS dental provision is generally less than would be needed to provide basic dental care. The interpretation of outputs needs to take account of edge-effects near to Scotland and Wales. Possible improvements include the inclusion of other modes of travel and the exclusion of the population that does not want to access NHS care.

## Introduction

Within England, residents are entitled to access National Health Service (NHS)-provided dental care. However, finding a dentist willing to provide such care to existing or new patients is increasingly difficult [1]. This has led to the formation of what some are calling ‘dental deserts’ [2]. In a recent patient survey [3], of the 53% of patients who had tried to book an NHS appointment, around 75% were successful. This success rate, however, fell to just 33% for those who had not previously been seen by the practice. Of those who did not try to get an appointment, 21% said they did not need one, but 22% said they did not even try because they thought they would not be able to get an appointment. With the need for dental treatments conditioned by socioeconomic status [4, 5], this deficiency in provision can be particularly impactful on some vulnerable communities [6]. To try and understand the nature of these differences in access to health care, Penchansky and Thomas [7] describe five dimensions, three are aspatial, ‘Affordability’, ‘Acceptability’, and ‘Accommodation’, while two, ‘Availability’ and ‘Accessibility’, are spatial. The affordability aspect covers the ability of the patient to pay for the care, with a simple NHS check-up costing around £25, and further treatments costing either £75 or £300 (although certain patients are exempt [8]). Which of these two treatment costs applies is determined by the type of work and not the amount of work. Accommodation is about the configuration of the provider’s offer and how easy it is for a patient to navigate [9], which could include the practice offering out-of-hour appointments on week nights or weekends, meaning that patients do not need to take time off work to attend. Acceptability encompasses the ambiance of the practice [10], including its welcoming atmosphere for patients and patient loyalty, where a patient will move between practices to follow a particular dentist. The remaining two dimensions are spatial. Availability captures the capacity of the provision at a location, and accessibility refers to the ease of travel to access the provider. These two dimensions are the focus of this study.

Previous studies have measured availability using indicators such as the number of NHS dental practices or dentists available in a neighbourhood [11], usually expressed as a provider-to-population ratio [12]. Accessibility is measured in most studies as a distance to the nearest provider, or more generally, the number of providers reachable within a set catchment [13]. Hauser’s [14] table 3 lists the disadvantages of using these indicators to measure spatial access. The ratio-type indicators suffer from the problem of containerization, assuming that the demand occurs within the same geographical neighbourhood as the provision—a provider cannot serve a patient outside the neighbourhood and a patient cannot use a provider located outside the neighbourhood. In some health contexts, this is a reasonable assumption, but not in the case of NHS dentistry. A dentist can treat anyone, irrespective of their home location, and a patient can visit any dentist willing to treat them. Catchment measures do not account for supply competition. Such competition occurs when a practice is within the catchment of one or more neighbourhoods, with an assumption that the practice provision is equally available for all such neighbourhoods. This is not the case; the finite and fixed provision cannot be duplicated.

**Table 1.** Distribution statistics for IMD

	SDI (availability)			SPAI (accessibility)		
	First quartile	Median	Third quartile	First quartile	Median	Third quartile
Highest deprivation	1376	1571	1815	1.23	1.42	1.62
High deprivation	1290	1504	1745	1.11	1.31	1.53
Middle deprivation	1275	1481	1739	1.01	1.23	1.46
Low deprivation	1269	1477	1737	1.00	1.22	1.44
Lowest deprivation	1218	1415	1681	0.98	1.17	1.38

**Table 2.** Distribution statistics for urban/rural classification

Classification	SDI (availability)			SPAI (accessibility)		
	First quartile	Median	Third quartile	First quartile	Median	Third quartile
Major urban	1298	1521	1553	1.20	1.40	1.61
Minor urban	1586	1708	1734	1.42	1.54	1.68
Urban city/town	1233	1425	1467	1.04	1.22	1.42
Rural town	1354	1593	1635	0.88	1.10	1.34
Rural village	1335	1556	1616	0.71	0.93	1.18

**Table 3.** Reductions in spatial density and accessibility after the removal of BUPA dental practice

LSOA	SDI (availability)		SPAI (accessibility)		Change in rank	
	With	Without	With	Without	SDI	SPAI
E01014486	1358.4	1279.1	1.2891	1.1788	2990	4565
E01033366	1472.5	1182.4	1.3329	1.0854	10 671	11 921
E01014488	1328.6	1147.1	1.2403	1.0487	5793	7423
E01014489	1255.3	1071.8	1.1876	0.9856	4590	7094
E01033348	1260.3	1182.9	1.2044	1.1023	2329	4072
E01033347	1481.5	1187.7	1.4050	1.1050	10 890	12 078
E01014485	1347.6	1163.4	1.2495	1.0530	6180	7686

To address the lack of realism in these methods, various modelling approaches under the term Floating Catchment Areas (FCAs) have been developed and used in the literature [15]. At their simplest, the two-step FCA (2SFCA) proceeds as follows: Step 1 calculates a provider demand ratio within a catchment, which is the number of patients per provider that they are able to treat. In Step 2, these provider demand ratios that are within a catchment of the patient’s location are summed. This final sum represents a Spatial Accessibility Index (SPAI) for the neighbourhood, where a higher value indicates a higher number of provider locations (or provision), or lower demand, or higher proximity between provider and patient locations.

Adaptations of this approach are possible to deal with distance decay, supply competition, and relative versus absolute travel distances. Once again, table 3 of Hauser [14] outlines the advantages of these varying degrees of sophistication. Such approaches have been used to assess the accessibility of various types of providers, including bank branches [16], public transit [17], and fire stations [18]. They have also been extensively used in the health domain [19–21], and specifically in relation to access to dentistry for the general [22] and more specific populations [23]. These methods have also served to inform quality of care [24] and as what-if tools to evaluate the impact of retirements in provision [25].

The motivation for this study is to show how the availability and accessibility of NHS dentistry vary across England, illustrating their potential to identify dental deserts. These indices are also characterized by the type of neighbourhood, helping to reveal any systemic variations in provision. Finally, the study demonstrates how it can be used to assess changes in provision if the level of demand or capacity is varied; if new practices open for NHS patients; or if existing practices close. This study achieves this by applying the Modified Huff Variable Three Step Floating Catchment Area (MHV3SFCA) [26] modelling approach to measure the accessibility to NHS dental care in England during the financial year 2022–23. Provider provision is measured in Units of Dental Activity (UDA) [3], demand is measured using the 2021 Census population at the geography of Lower Super Output Area (LSOA) [27], and the travel between LSOAs and providers is measured by car journey times [28].

## Methods

The MHV3SFCA modelling approach addresses several concerns related to the application of FCA methods [26]. It incorporates Huff probabilities and a travel time decay function to consider competition for dental services, where the demand from a specific location increases with the provision at the practice and decreases with distance. Unlike fixed catchment travel time values, this approach uses variable travel times, taking into account location-specific travel times to ensure access to a specific number of reachable sites. Additionally, it considers differences in absolute travel times rather than relative times, and the overall demand is independent of provision. The model produces two outputs: a Spatial Density Index (SDI), which measures availability, and is similar to a ratio of provision to population (here in units of UDAs per 1000 people), and a SPAI, which measures a spatial accessibility score.

The MHV3SFCA approach requires a number of parameters, primarily associated with the travel time decay function. Of most importance is probably the value of *Q*, the number of reachable locations. A value of *Q* of 1 restricts the choice to just the nearest location, while a high value would include many potentially impractical locations. In an online survey of residents of rural Lincolnshire, it was found that patients contacted upwards of 10 practices to try and find an appointment [29]; since such rural locations would probably be the most challenging scenario, here, a conservative value for *Q* of 10 is used. Given the use of *Q*, the other distance decay parameters of *d*_max_ and *f*(*d*_max_) lose some of their importance, but to inform these choices, Supplementary Fig. S1 shows the distribution of the minimum, mean, and furthest travel times. The median travel time between dentists and LSOAs is 28 min, and the mean is 29, so the value used here for *d*_max_ is 30 min (Supplementary Fig. S2 shows the profile of the decay function for various values of *d*_max_). The function value at *d*_max_ is the final parameter, here set at 0.05 [Supplementary Fig. S3 shows the profile of the decay function for various values of *f*(*d*_max_)]. This combination of values means that a travel time of 30 min will have a weight of 0.05; however, since only the 10 closest locations are considered, these weights are only important for the 10 closest locations.

The demand population is the usual resident population of the LSOA counted at the 2021 Census. Each LSOA comprises between 400 and 1200 households and has a population between 1000 and 3000 persons [27]. The geographic location of this demand is the population-weighted centroid of the LSOA which reflects the distribution of population within its boundaries. The provision is measured using UDAs. Band 1 work (mainly examinations and x-rays) consumes 1 UDA, the band 2 treatments (mainly fillings and extractions) consume between 3 and 7 UDAs, while the most complex treatments (dentures and bridges) consume 12 UDAs. The allocation of UDAs to each practice is published by the NHS [30], and the figures for the 2022–23 financial year are used, with a median allocation of 10 740 UDAs and a mean of 13 277. There is an expectation that practices will deliver at least 95% of their contracted UDAs. The location of practices is determined by their postcode, where provided.

The final piece of data is the journey times between practices and LSOAs. There are 6506 dental practices under contract to deliver NHS dentistry and 33 754 LSOAs in England, requiring the calculation of nearly 220 million journey time combinations. Many of these journey times will be long and impractical, so instead here only journey times where the crow fly distance is <32 km are calculated, with those times beyond this limit set to 120 min (Jo *et al*. [13] set a maximum buffer size threshold of 10 km to identify dental practices). The binary **B** matrix of Ref. [14] is constructed so that travel to dental practices beyond the 10th closest will be ignored in the calculation. However, there are two exceptions to this case. First, given that the travel times are calculated to one decimal place, there are 6212 LSOAs (18%) with a tie for the 10th fastest journey time, meaning they have a *Q* value >10. Second, a consequence of the 32 km threshold is that for 32 LSOAs (0.1%) located along the Scottish and Welsh borders, there are <10 practices reachable, meaning a *Q* value <10 is used for such LSOAs.

The models are calculated using the R [31] code provided in Ref. [14].

The MHV3SFCA approach allows for the exploration of various what-if scenarios using these data. If the demand population changes at a location (through population decline/growth) or the provision at a practices changes, then the work required to evaluate the impact of these is trivial through changes to the population or UDA counts. If a practice or a demand is to be removed from the model, this can be relatively easily achieved by removing the provision or demand and the appropriate row (for removing a dental practice) or column (for removing a population demand) from the travel time matrix. There is then a need to recalculate the (now smaller) **B** matrix of Ref. [14] before the re-modelling. If there is a new practice or population demand, then there is the need for an additional piece of work to calculate the journey times from/to the new location. Then, a calculation of the new rows or columns in an updated version of this slightly larger **B** matrix is required.

## Results

In the 2021 Census, the population of England was 56 489 800 and during the 2022–23 financial year, the NHS contracted 86 381 814 UDAs with 6506 geo-locatable practices (99.3% of all UDAs). This provides a population-level coverage of just 1.53 UDAs per capita, which is insufficient to provide the recommended two examinations per year, let alone any treatments.

**Figure 1.**
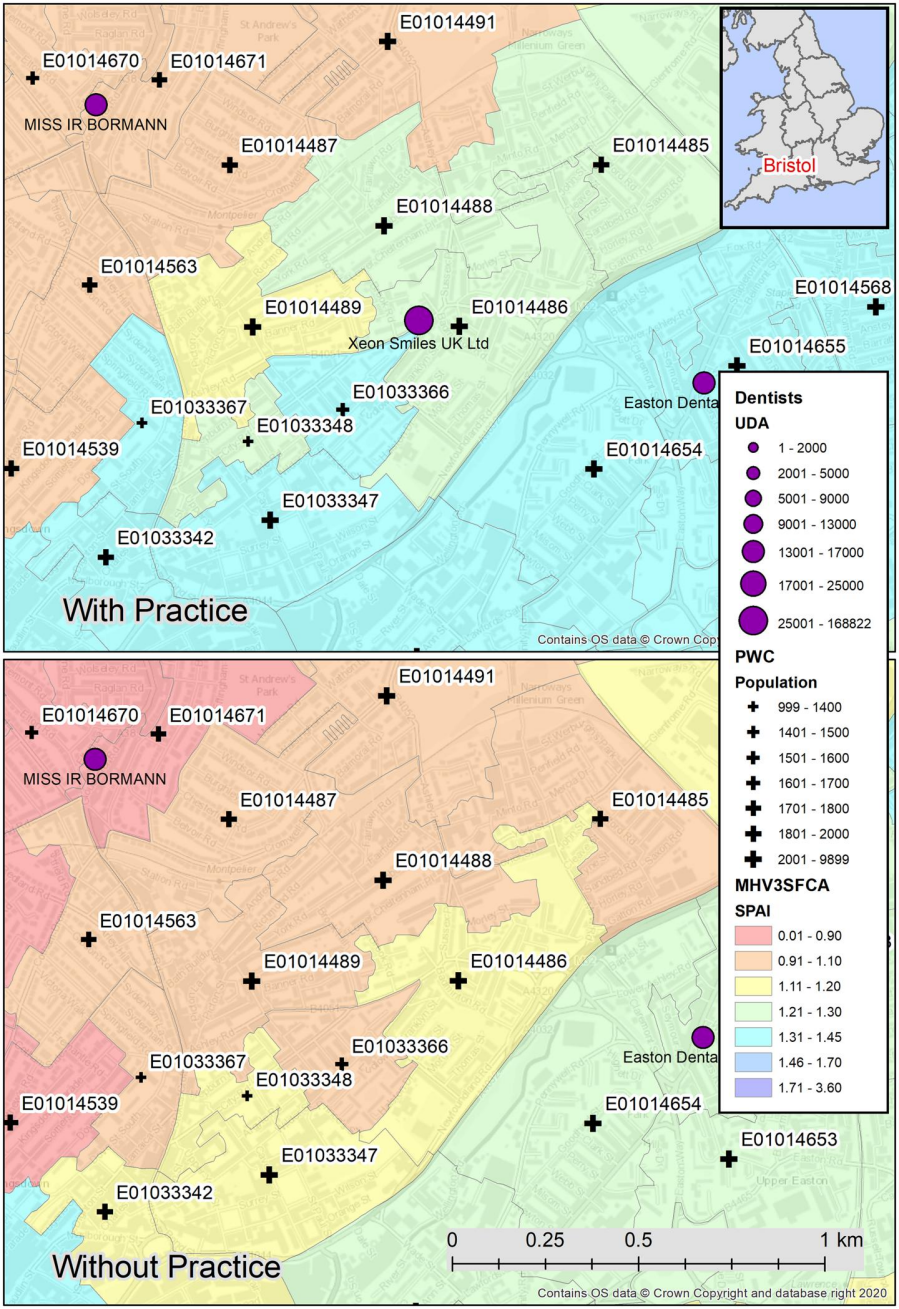
Change in SPAI after closure of dental practice in St Pauls, Bristol, UK.

Using the outputs from the MHV3SFCA model, it is possible to map how the availability and accessibility of NHS dentistry varies by LSOA within England (see Fig. 1 later for such an example). It is also possible to examine how the accessibility measures vary by the characteristics of the LSOA. Here, an index of multiple deprivation (IMD) [32] and urban/rural classification [33] is carried over from 2011 Census LSOAs. Table 1 shows the distributional statistics for availability and accessibility for the IMD, while Table 2 shows the same for the urban/rural classification.

In Table 1, there is a clear positive gradient with deprivation; areas that are more deprived have greater SDI availability of care (the areas with the highest deprivation have on average 1571 UDAs available per 1000 population) and also have higher SPAI accessibility. For urban/rural locations, minor urban conurbations have the highest density of provision (1708 UDAs per 1000 population) and the best accessibility. Rural locations also have high density of provision (possibly from having a generally smaller population than urban areas), but the accessibility for rural locations is poor.

The results presented above establish a base case from which it is possible to explore various what-if type scenarios. Here, a scenario based on a recent event in Bristol is used [34]. In June 2023, the BUPA Dental Care practice (trading as Xeon Smiles UK Ltd) on Ashley Court in the St Pauls area of Bristol closed down. In the 2022–23 NHS Business Agency data [30] for that provider, 31 772 UDAs were contracted for. To illustrate the impact of this closure, Table 3 shows the level of dental provision available (SDI) and accessibility (SPAI) for surrounding LSOAs, with and without this provision. The change in ranking of the LSOA among all LSOAs is also provided.

In each of these LSOAs, the availability SDI typically reduces from 1300 UDAs per 1000 population to around 1200 UDAs. There are also significant reductions in the SPAI, as illustrated by how far down the ranking each LSOA falls. The home LSOA of the practice (E01014486) is actually one of the least impacted by the loss of this provision, but the knock-on effects to neighbouring LSOAs are significant, as seen in the before and after map of the St Pauls locale in Fig. 1. In this map, it is clear that the level of provision reduces both locally and at some distance from the location of the practice, indicating greater supply competition at the remaining practices when this practice is removed. Fortunately, according to a February 2024 news story, some level of provision has now been restored to this community.

## Discussion

This study has reported on a modelling approach that attempts to realistically measure the availability of and accessibility to dental practices in England that offer NHS-funded care. The results show that access is actually best where it is most needed, in deprived neighbourhoods. Other studies have also shown such a gradient, with Jo *et al*. [35] reporting the best access in the most deprived decile in Scotland and Wales, and the second-best access in Northern Ireland. The results here regarding access by the urban/rural nature of the neighbourhood also confirm the results reported in other studies [13].

The utility of the MHV3SFCA modelling approach to explore various supply and demand-side what-if scenarios is also demonstrated. This is especially important since the two largest UK political parties have made commitments to make modest increases in NHS provision. The Conservative government has committed to the creation of 240 extra practices [36], but with no detail on the level of provision (i.e. how many UDAs) or where they will be located. Similarly, the Labour Party has committed to the addition of 700 000 more NHS appointments (presumably the equivalent of 700 000 UDAs) [37], but again with no detail on where they are to be made available. This model can help here. It can be used to identify neighbourhoods where access is currently poor, or more targetedly, neighbourhoods of a particular type where access is currently poor (i.e. deprived). It can alternatively be used to optimally identify a finite number of locations where provision can be increased to achieve the greatest increase in overall availability and accessibility. However, it must be mentioned that these figures for extra practices and care are modest against the scale of increased demand and that the level of central government control is limited, since dental practices are private businesses with the freedom to do business where they want [38].

The population geography adopted in this study is a compromise. From the 2021 Census, counts of population are available at the smaller geographies of postcode (1 293 858 postcodes in England, with a median population of 33) and Output Area (OA) (178 605 OAs in England, with a median population of 306). However, such locations impose an increased computational burden, especially in the calculation of journey times and may produce noisy results that do not reflect the more smoothed nature of accessibility. However, the LSOAs adopted here may still be too granular for planning and optimization tasks as described above, with still the possibility of variability in accessibility over a short distance. It would be unwise to invest in a new practice for just a few neighbouring LSOAs that had poor accessibility. Instead, it is possible to aggregate the SDI and SPAI here to larger geographies, such as Middle Super Output Areas [27] or Parliamentary constituencies. This approach allows the use of data calculated at the granular level of LSOA to be assessed at a geographically more strategic level.

One aspect of geography that is difficult to overcome is edge effects. There are LSOAs in Northumbria near the Scottish border and in Hereford near the Welsh border, which have particularly poor accessibility. This is partly due to their rural nature, but also because no account is taken here of dental provision in Scotland or Wales, which English residents can access. In Scotland, the contract with dentists does not use UDAs; instead, practices are paid an annual amount for each registered patient, and patients are entitled to free examinations but pay 80% of treatment costs, capped at just under £400. It is difficult to see how this arrangement could be translated into the English UDA context. In Wales, a hybrid system operates, where practices can opt to use a UDA approach or one that has a greater emphasis on preventative care, targeting those with greater need, and the achievement of various target metrics. By the summer of 2023, 78% of dental practices in Wales had adopted this non-UDA approach. Again, this divergence makes it difficult to accommodate Welsh practices in this model. It is, however, possible to use the MHV3SFCA approach to assess access to NHS dentistry within Scotland and Wales, but this must be done while recognizing the discontinuity along the English border.

For further work, it might be insightful to see if the use of smaller geographies such as postcodes or OAs produces additional local insight. Other studies have also used a multi-modal approach to working out the travel distances or times, calculating two SPAIs or as Hauser [14] does, creating a hybrid travel matrix which uses the quickest journey time, irrespective of mode of travel, between each provider and population. Investigation of what patients may do when access to NHS dentistry is difficult could be considered, particularly as the General Practice Dental survey indicates that they may seek more expensive local private dental provision [3]. One solution is to try to incorporate private dental provision, another is to relax the assumption that the demand for dental care is captured through the size of the population. As the General Practice dental survey identifies, a sizeable 29% of survey respondents preferred private care to NHS care [3]. If there was a mechanism to assess the spatial variability of this 29% of the population at the LSOA geography, then they could be discounted for modelling purposes.

## Supplementary Material

ckae099_Supplementary_Data

## Data Availability

The data used for this study are available and downloadable from public data sources. NHS payments to dentists’ data are available from https://www.nhsbsa.nhs.uk/dental-data/nhs-payments-dentists and 2021 Census counts from https://www.nomisweb.co.uk/sources/census_2021. Key points Availability of NHS dental provision varies by neighbourhood and neighbourhood type. Accessibility to NHS dental provision varies by neighbourhood and neighbourhood type. A method of using the model to explore changes in dental provision and population on NHS density provision is described. Use of the model to help shape the implementation of additional NHS dental resources, through new practice locations or extra provision.
