# Supplementary material for: Spatial disparities in access to NHS dentistry: a neighbourhood-level analysis in England
Source: Eur J Public Health. 2024 Jun 22;34(5):854–9. doi: 10.1093/eurpub/ckae099 (PMC11430962; doi:10.1093/eurpub/ckae099)
Supplement: ckae099_Supplementary_Data [file ckae099_supplementary_data.docx]

Spatial Disparities in Access to NHS Dentistry: A Neighbourhood-Level Analysis in England.

Stephen D Clark

Supplimentary material


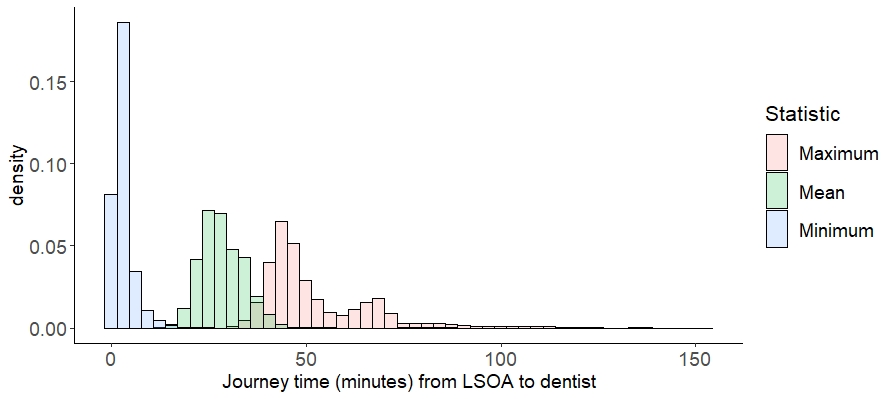


Figure S1 : Distribution of travel time to closest (minimum), average (mean) and furthest (maximum) dental practice


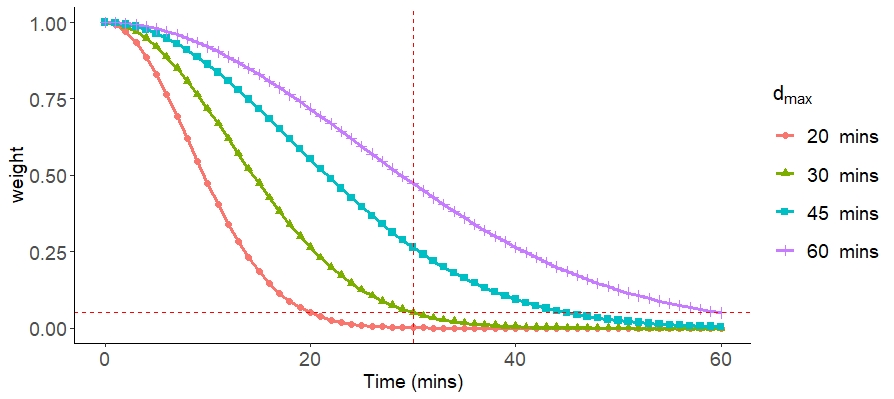


Figure S2 : The profile of the decay function for a range of values of d_max_, with 30 minutes and a weight of 0.05 indicated with red dashes.


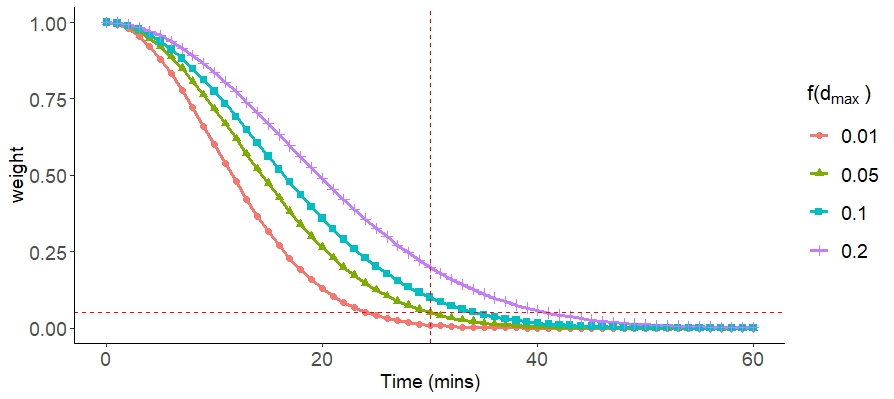


Figure S3 : The profile of the decay function for a range of values of f(d_max_), with 30 minutes and a weight of 0.05 indicated with red dashes.
